# Supplementary material for: Prototropy, Intramolecular Interactions, Electron Delocalization, and Physicochemical Properties of 1,8-dihydroxy-9-anthrone—DFT-D3 Study of Substituent Effects
Source: Molecules. 2023 Jan 1;28(1):344. doi: 10.3390/molecules28010344 (PMC9822037; doi:10.3390/molecules28010344)
Supplement: Supplementary file 1 [file molecules-28-00344-s001.zip › molecules-1895215-supplementary.pdf]

Table S1 Bond lengths in crystal structures of 1,8-dihydroxy-9-anthrone derivatives

Refcode	*HOC1*	*HOC8*	*C9-O*	*C1-O*	*C8-O*	*4a-9a*	*8a-10a*	*9-9a*	*9-8a*	*10-4a*	*10-10a*
BUFWAD	109.537	109.511	1.253	1.345	1.35	1.409	1.412	1.455	1.456	1.483	1.487
BUFWAD	109.532	109.426	1.253	1.352	1.341	1.418	1.407	1.461	1.452	1.483	1.481
SOSCUB	105.16	105.427	1.271	1.352	1.353	1.412	1.415	1.445	1.455	1.526	1.527
YOXJAZ	109.584	109.459	1.253	1.342	1.339	1.423	1.414	1.475	1.46	1.508	1.488
YOXJAZ	109.987	109.533	1.241	1.339	1.348	1.407	1.408	1.48	1.464	1.499	1.477
YOXJAZ	109.074	109.409	1.243	1.339	1.337	1.402	1.406	1.475	1.461	1.516	1.485
YOXJAZ	109.305	109.599	1.249	1.34	1.341	1.422	1.412	1.477	1.461	1.497	1.482
ANTHLN10	104.516	104.94	1.261	1.356	1.354	1.413	1.409	1.45	1.448	1.492	1.492
ATEQIZ	111.282	104.384	1.255	1.342	1.349	1.42	1.415	1.462	1.473	1.496	1.487
ATEQOF	102.827	99.644	1.264	1.351	1.338	1.422	1.413	1.451	1.453	1.478	1.488
AVERUF	105.474	109.661	1.255	1.351	1.346	1.404	1.409	1.455	1.452	1.475	1.483
AXELAR	109.456	109.464	1.26	1.347	1.348	1.415	1.411	1.466	1.46	1.491	1.491
BEJZEV	106.961	102.221	1.254	1.36	1.353	1.407	1.398	1.457	1.452	1.508	1.503
BEJZEV11	105.343	103.443	1.257	1.337	1.354	1.406	1.41	1.445	1.467	1.507	1.496
BEJZEV12	109.457	109.48	1.256	1.352	1.351	1.407	1.404	1.465	1.462	1.51	1.507
BIGJIN	109.467	109.433	1.277	1.353	1.348	1.432	1.454	1.475	1.443	1.495	1.467
BIKLEN	105.122	107.552	1.295	1.352	1.359	1.45	1.419	1.417	1.457	1.452	1.483
BOLPEX	101.602	115.778	1.242	1.35	1.338	1.404	1.397	1.462	1.459	1.491	1.49
CARMYC11	94.94	114.611	1.255	1.343	1.337	1.396	1.391	1.457	1.47	1.475	1.497
DHANQU01	109.486	109.486	1.248	1.343	1.343	1.406	1.406	1.447	1.447	1.478	1.478
DHANQU02	109.518	109.506	1.249	1.332	1.348	1.415	1.412	1.464	1.459	1.494	1.481
DHANQU03	109.504	109.447	1.25	1.344	1.324	1.42	1.4	1.43	1.471	1.505	1.451
DHANQU04	109.461	109.574	1.241	1.346	1.346	1.401	1.413	1.474	1.467	1.506	1.491
DHANQU04	109.451	109.501	1.251	1.335	1.333	1.413	1.408	1.471	1.468	1.482	1.487
DHANQU05	109.455	109.527	1.281	1.359	1.35	1.409	1.409	1.434	1.452	1.498	1.472
DHANQU05	109.471	109.57	1.259	1.355	1.344	1.413	1.418	1.49	1.449	1.461	1.484
DHANQU05	109.43	109.42	1.26	1.346	1.349	1.419	1.411	1.462	1.465	1.488	1.485
DHANQU05	109.425	109.518	1.253	1.362	1.341	1.421	1.427	1.444	1.481	1.485	1.489
DHANQU06	109.463	109.458	1.253	1.331	1.34	1.421	1.413	1.459	1.47	1.49	1.484
DHANQU07	109.413	109.413	1.225	1.361	1.361	1.386	1.386	1.463	1.463	1.496	1.496
DHANQU08	109.6	109.49	1.257	1.352	1.355	1.411	1.401	1.527	1.395	1.524	1.477
DIPFAM	106.049	104.375	1.264	1.345	1.349	1.412	1.415	1.46	1.451	1.492	1.49
DIPFEQ	106.821	104.934	1.264	1.344	1.355	1.418	1.415	1.439	1.463	1.495	1.485
DIPFEQ	102.47	89.368	1.263	1.354	1.35	1.422	1.415	1.441	1.459	1.495	1.49
EGINIU	109.573	109.35	1.259	1.347	1.354	1.4	1.417	1.457	1.449	1.521	1.533
EGINIU	109.46	109.495	1.268	1.361	1.345	1.415	1.431	1.451	1.418	1.54	1.519
ETANIY	107.064	107.547	1.256	1.34	1.349	1.413	1.411	1.457	1.459	1.476	1.493
ETANIY01	109.388	109.472	1.268	1.345	1.347	1.427	1.416	1.437	1.452	1.475	1.484
ETANOE	107.922	109.428	1.268	1.352	1.353	1.422	1.418	1.447	1.453	1.477	1.478
ETAPIA	109.428	109.491	1.265	1.346	1.348	1.396	1.412	1.454	1.457	1.484	1.477
FEDMEH	109.478	109.499	1.26	1.347	1.345	1.413	1.413	1.441	1.463	1.49	1.477
FEPKOA	110.207	102.124	1.27	1.354	1.364	1.435	1.427	1.429	1.445	1.485	1.487
GACPAE	109.465	109.473	1.265	1.357	1.361	1.416	1.411	1.459	1.449	1.516	1.519
GACPEI	109.492	109.462	1.268	1.354	1.36	1.418	1.414	1.457	1.45	1.513	1.513

HIVXIU	109.434	109.355	1.269	1.357	1.356	1.423	1.412	1.423	1.481	1.479	1.445
HOLGEV	109.512	109.438	1.274	1.358	1.354	1.417	1.417	1.439	1.447	1.487	1.49
JUKREM	100.299	108.424	1.24	1.354	1.344	1.406	1.435	1.477	1.442	1.483	1.489
KIPGIC	108.17	105.825	1.263	1.344	1.348	1.411	1.414	1.45	1.463	1.488	1.483
KIPGOI	103.622	108.109	1.261	1.353	1.341	1.417	1.413	1.46	1.451	1.486	1.481
KIPGUO	107.235	106.927	1.26	1.343	1.353	1.413	1.403	1.458	1.465	1.484	1.487
KIPHAV	109.404	109.431	1.265	1.356	1.348	1.418	1.414	1.438	1.468	1.479	1.479
KIPHAV	109.476	109.478	1.26	1.35	1.354	1.41	1.414	1.442	1.474	1.491	1.48
KIPHAV	109.401	109.509	1.257	1.355	1.353	1.404	1.413	1.451	1.462	1.485	1.472
KIPHAV	109.475	109.465	1.254	1.346	1.347	1.409	1.405	1.456	1.466	1.475	1.482
KIPHEZ	109.45	109.47	1.26	1.346	1.358	1.418	1.412	1.448	1.465	1.495	1.489
KIPHEZ01	109.447	109.486	1.253	1.359	1.357	1.418	1.421	1.446	1.453	1.48	1.497
KIPHOJ	102.909	106.631	1.264	1.349	1.35	1.418	1.421	1.464	1.451	1.485	1.485
LARVEG	109.485	109.466	1.253	1.339	1.347	1.408	1.423	1.448	1.462	1.482	1.486
LETNII	109.459	109.441	1.269	1.347	1.349	1.415	1.427	1.44	1.45	1.458	1.487
MANVEF	109.267	104.535	1.254	1.344	1.343	1.417	1.412	1.46	1.465	1.488	1.485
MEMWUX	109.445	109.498	1.263	1.35	1.351	1.416	1.404	1.447	1.461	1.486	1.491
MIWVEV	109.399	101.043	1.263	1.341	1.354	1.415	1.41	1.422	1.449	1.527	1.516
NABSAK	107.573	120.87	1.26	1.354	1.338	1.415	1.423	1.451	1.442	1.487	1.498
NIFMOH	112.814	99.191	1.256	1.341	1.357	1.404	1.418	1.464	1.449	1.497	1.501
NIFMOH	103.639	109.872	1.255	1.346	1.349	1.415	1.413	1.467	1.452	1.484	1.491
NIFMUN	99.252	103.432	1.27	1.363	1.349	1.421	1.417	1.454	1.456	1.505	1.506
NIFMUN	110.977	101.977	1.264	1.35	1.341	1.412	1.408	1.458	1.45	1.5	1.514
NUQPOF	106.685	107.712	1.232	1.317	1.317	1.396	1.396	1.473	1.474	1.493	1.491
OGEROI	103.81	107.167	1.26	1.346	1.347	1.413	1.417	1.467	1.449	1.485	1.495
OGEROI	103.089	106.161	1.259	1.353	1.345	1.415	1.417	1.462	1.444	1.488	1.492
PIRFIH	109.433	109.427	1.228	1.318	1.329	1.401	1.389	1.481	1.477	1.494	1.496
PIRFON	109.434	109.428	1.248	1.322	1.32	1.407	1.401	1.47	1.475	1.489	1.497
QEGXUV	97.681	112.04	1.271	1.348	1.36	1.41	1.419	1.452	1.435	1.522	1.517
QEGXUV	107.465	107.364	1.258	1.353	1.344	1.415	1.382	1.444	1.451	1.532	1.521
RAYXEX	109.49	102.877	1.268	1.339	1.358	1.406	1.412	1.442	1.46	1.486	1.48
RAYXIB	109.444	109.426	1.263	1.35	1.357	1.413	1.408	1.467	1.449	1.487	1.503
RAYXOH	109.494	109.502	1.265	1.345	1.349	1.412	1.416	1.452	1.458	1.492	1.482
RAYXUN	109.529	109.49	1.277	1.354	1.343	1.382	1.435	1.52	1.386	1.498	1.469
RAYYAU	105.304	104.176	1.258	1.353	1.346	1.417	1.415	1.46	1.455	1.483	1.491
RAYYEY	109.479	109.475	1.26	1.347	1.346	1.411	1.417	1.471	1.448	1.488	1.486
RHODON	104.329	104.598	1.258	1.349	1.344	1.428	1.425	1.445	1.474	1.444	1.485
RHODON	111.589	104.187	1.259	1.31	1.34	1.435	1.417	1.451	1.462	1.457	1.482
ROFJUT	109.399	109.548	1.261	1.347	1.326	1.411	1.425	1.459	1.45	1.493	1.483
SAPQAB	109.633	106.295	1.256	1.345	1.348	1.423	1.402	1.453	1.463	1.46	1.487
SOHXAO	105.723	107.493	1.275	1.366	1.347	1.404	1.403	1.46	1.458	1.479	1.516
SOHXAO01	106.512	105.851	1.254	1.346	1.348	1.405	1.413	1.465	1.465	1.492	1.484
SUQBOX	106.437	107.247	1.261	1.354	1.352	1.413	1.418	1.461	1.456	1.504	1.505
SUQCAK	107.267	107.037	1.265	1.357	1.357	1.412	1.417	1.456	1.459	1.505	1.502
TEVVOG	109.447	109.43	1.266	1.355	1.358	1.413	1.404	1.455	1.45	1.48	1.49
TIDCIS	104.896	105.889	1.242	1.337	1.331	1.407	1.419	1.462	1.465	1.49	1.492

TIDCIS	103.062	106.093	1.244	1.337	1.339	1.412	1.419	1.464	1.465	1.477	1.482
TUPKUK	109.474	109.472	1.251	1.341	1.346	1.405	1.415	1.465	1.458	1.489	1.485
VANCET	104.99	106.825	1.26	1.346	1.36	1.407	1.407	1.463	1.461	1.516	1.521
VERSIC	109.104	106.558	1.266	1.356	1.35	1.42	1.421	1.44	1.459	1.492	1.47
VURHEV	122.56	130.412	1.282	1.385	1.368	1.433	1.446	1.391	1.475	1.539	1.504
WOKYIH	111.12	106.102	1.264	1.351	1.347	1.403	1.41	1.46	1.459	1.523	1.53
WOKYIH	107.847	109.208	1.258	1.345	1.344	1.413	1.414	1.461	1.461	1.486	1.484
XABSAX	106.374	109.337	1.267	1.354	1.344	1.412	1.415	1.452	1.456	1.475	1.486
XABSAX	104.021	106.951	1.266	1.353	1.344	1.411	1.412	1.459	1.45	1.474	1.48
XABSAX	104.844	108.132	1.269	1.352	1.344	1.411	1.416	1.455	1.452	1.476	1.475
XABSAX	112.689	106.428	1.239	1.334	1.345	1.417	1.418	1.465	1.463	1.499	1.497
XABSAX	110.244	107.487	1.243	1.332	1.343	1.413	1.415	1.47	1.458	1.492	1.487
XABSAX	110.605	105.168	1.245	1.34	1.342	1.413	1.416	1.464	1.467	1.484	1.491
YOKHIP	108.097	107.612	1.254	1.342	1.344	1.403	1.397	1.449	1.462	1.481	1.5
ZISQUQ	108.853	107.445	1.26	1.34	1.333	1.436	1.408	1.452	1.455	1.449	1.494
VUMDIT	109.448	109.453	1.257	1.34	1.348	1.402	1.4	1.46	1.467	1.491	1.485
VUMDIT	109.463	109.507	1.258	1.328	1.345	1.412	1.416	1.458	1.463	1.485	1.477
YUKPUS	109.458	109.46	1.26	1.342	1.348	1.415	1.41	1.446	1.467	1.48	1.479

Table S1. c.d.

Refcode	*1-9a*	*1-2*	*2-3*	*3-4*	*4-4a*	*8-8a*	*7-8*	*6-7*	*5-6*	*5-10a*	*HOC8C8a*	*HOC1C9a*
BUFWAD	1.398	1.401	1.346	1.403	1.385	1.406	1.395	1.373	1.394	1.38	8.421	0.84
BUFWAD	1.403	1.398	1.365	1.396	1.378	1.404	1.394	1.36	1.391	1.378	-0.282	-2.569
SOSCUB	1.419	1.382	1.381	1.399	1.379	1.411	1.398	1.387	1.397	1.383	2.001	-2.089
YOXJAZ	1.415	1.382	1.375	1.411	1.396	1.409	1.389	1.37	1.397	1.381	-0.855	1.722
YOXJAZ	1.416	1.383	1.375	1.393	1.402	1.403	1.396	1.389	1.375	1.372	0.197	-3.186
YOXJAZ	1.412	1.392	1.37	1.411	1.4	1.406	1.401	1.378	1.384	1.387	4.934	-14.838
YOXJAZ	1.404	1.396	1.387	1.406	1.392	1.415	1.39	1.381	1.389	1.387	-8.19	0.212
ANTHLN10	1.409	1.379	1.367	1.379	1.377	1.414	1.384	1.375	1.379	1.38	-1.946	-9.689
ATEQIZ	1.417	1.4	1.372	1.414	1.368	1.406	1.4	1.405	1.395	1.386	8.809	1.882
ATEQOF	1.402	1.389	1.387	1.403	1.385	1.411	1.396	1.403	1.391	1.38	2.744	-1.806
AVERUF	1.408	1.388	1.381	1.388	1.39	1.404	1.391	1.388	1.392	1.372	-2.792	5.859
AXELAR	1.414	1.406	1.386	1.405	1.387	1.42	1.401	1.396	1.4	1.383	-0.271	0.344
BEJZEV	1.418	1.392	1.367	1.382	1.389	1.426	1.384	1.354	1.401	1.383	-3.537	-5.393
BEJZEV11	1.411	1.38	1.361	1.39	1.378	1.41	1.375	1.372	1.385	1.391	-0.121	-7.876
BEJZEV12	1.413	1.389	1.375	1.387	1.388	1.418	1.382	1.379	1.388	1.386	1.569	-0.66
BIGJIN	1.387	1.437	1.387	1.468	1.395	1.418	1.463	1.432	1.442	1.407	-4.538	-6.498
BIKLEN	1.402	1.387	1.39	1.467	1.407	1.401	1.391	1.391	1.388	1.391	1.81	0.71
BOLPEX	1.399	1.384	1.373	1.386	1.381	1.412	1.39	1.371	1.39	1.383	-15.103	-12.024
CARMYC11	1.401	1.425	1.365	1.419	1.409	1.425	1.392	1.345	1.4	1.386	5.772	18.197
DHANQU01	1.405	1.383	1.363	1.388	1.378	1.405	1.383	1.363	1.388	1.378	-1.598	-1.598
DHANQU02	1.405	1.387	1.392	1.389	1.38	1.402	1.4	1.373	1.434	1.386	1.204	-8.952
DHANQU03	1.416	1.364	1.334	1.433	1.358	1.42	1.392	1.392	1.319	1.385	-20.61	19.943
DHANQU04	1.402	1.396	1.379	1.402	1.371	1.396	1.409	1.367	1.397	1.387	4.728	-17.314
DHANQU04	1.411	1.409	1.369	1.389	1.383	1.413	1.402	1.351	1.398	1.376	5.321	-4.803

DHANQU05	1.417	1.39	1.366	1.407	1.39	1.396	1.413	1.352	1.373	1.394	-4.901	-5.258
DHANQU05	1.403	1.39	1.372	1.396	1.391	1.384	1.394	1.383	1.387	1.386	3.168	-7.55
DHANQU05	1.389	1.404	1.371	1.377	1.388	1.4	1.389	1.383	1.393	1.39	5.57	-2.786
DHANQU05	1.382	1.408	1.384	1.386	1.387	1.397	1.383	1.386	1.403	1.36	-2.829	-4.816
DHANQU06	1.413	1.398	1.37	1.393	1.378	1.41	1.401	1.372	1.392	1.385	2.136	-5.189
DHANQU07	1.412	1.373	1.285	1.439	1.389	1.412	1.373	1.285	1.439	1.389	12.544	12.544
DHANQU08	1.428	1.395	1.342	1.397	1.338	1.385	1.393	1.365	1.373	1.425	-15.538	29.666
DIPFAM	1.407	1.396	1.383	1.405	1.382	1.413	1.392	1.389	1.411	1.383	-0.339	0.534
DIPFEQ	1.417	1.387	1.394	1.405	1.372	1.404	1.394	1.387	1.407	1.38	9.381	0.898
DIPFEQ	1.412	1.39	1.391	1.402	1.377	1.412	1.386	1.393	1.407	1.376	-0.043	4.859
EGINIU	1.395	1.343	1.363	1.382	1.367	1.414	1.378	1.373	1.388	1.39	-9.435	-1.584
EGINIU	1.418	1.383	1.363	1.399	1.395	1.39	1.382	1.383	1.414	1.374	-0.214	7.544
ETANIY	1.413	1.402	1.372	1.397	1.38	1.41	1.393	1.375	1.412	1.371	2.31	2.265
ETANIY01	1.422	1.373	1.366	1.411	1.366	1.407	1.388	1.379	1.387	1.378	2.254	-4.867
ETANOE	1.408	1.378	1.379	1.404	1.376	1.408	1.389	1.38	1.387	1.373	1.807	0.387
ETAPIA	1.413	1.379	1.362	1.411	1.371	1.405	1.401	1.369	1.395	1.372	2.842	-4.467
FEDMEH	1.412	1.382	1.379	1.4	1.362	1.406	1.39	1.372	1.39	1.376	1.25	-0.211
FEPKOA	1.408	1.384	1.375	1.399	1.363	1.39	1.392	1.377	1.386	1.396	-8.814	-2.716
GACPAE	1.407	1.386	1.387	1.418	1.395	1.412	1.38	1.394	1.408	1.384	3.746	0.973
GACPEI	1.41	1.393	1.385	1.418	1.393	1.412	1.385	1.392	1.415	1.39	-3.45	-0.592
HIVXIU	1.42	1.356	1.393	1.394	1.376	1.373	1.386	1.387	1.364	1.395	0.189	0.636
HOLGEV	1.404	1.379	1.383	1.405	1.361	1.409	1.393	1.378	1.391	1.373	-1.347	6.4
JUKREM	1.396	1.397	1.389	1.391	1.374	1.397	1.399	1.365	1.372	1.393	1.075	24.271
KIPGIC	1.417	1.39	1.385	1.403	1.379	1.409	1.399	1.383	1.406	1.386	-1.446	-1.751
KIPGOI	1.405	1.393	1.383	1.394	1.38	1.418	1.396	1.369	1.402	1.386	-1.184	0.996
KIPGUO	1.417	1.386	1.384	1.404	1.363	1.409	1.394	1.388	1.393	1.392	-5.572	-3.372
KIPHAV	1.408	1.369	1.382	1.39	1.375	1.398	1.394	1.379	1.396	1.38	0.207	-0.489
KIPHAV	1.414	1.385	1.378	1.399	1.362	1.408	1.381	1.375	1.398	1.375	-8.929	1.335
KIPHAV	1.408	1.386	1.372	1.39	1.372	1.403	1.39	1.377	1.391	1.391	-1.092	4.917
KIPHAV	1.408	1.398	1.373	1.382	1.38	1.407	1.391	1.377	1.388	1.382	0.427	-0.892
KIPHEZ	1.414	1.388	1.396	1.411	1.38	1.405	1.396	1.387	1.4	1.385	3.99	-1.815
KIPHEZ01	1.416	1.386	1.365	1.404	1.37	1.415	1.37	1.387	1.375	1.377	0.583	-0.632
KIPHOJ	1.405	1.397	1.381	1.401	1.377	1.414	1.389	1.39	1.398	1.371	1.387	-6.23
LARVEG	1.408	1.388	1.374	1.389	1.378	1.399	1.389	1.383	1.413	1.397	10.142	2.458
LETNII	1.401	1.395	1.368	1.406	1.384	1.415	1.376	1.37	1.398	1.383	-0.858	-0.084
MANVEF	1.415	1.395	1.382	1.393	1.384	1.413	1.398	1.385	1.39	1.391	2.805	-1.375
MEMWUX	1.396	1.389	1.373	1.399	1.373	1.404	1.39	1.376	1.395	1.378	3.477	-0.664
MIWVEV	1.416	1.369	1.383	1.381	1.375	1.407	1.382	1.385	1.388	1.372	2.703	0.046
NABSAK	1.409	1.368	1.37	1.407	1.373	1.405	1.386	1.378	1.4	1.375	1.555	4.173
NIFMOH	1.4	1.396	1.387	1.403	1.397	1.404	1.406	1.389	1.406	1.379	-1.871	-7.62
NIFMOH	1.409	1.4	1.378	1.394	1.375	1.411	1.384	1.375	1.405	1.372	3.351	0.368
NIFMUN	1.405	1.384	1.391	1.396	1.368	1.415	1.387	1.394	1.385	1.383	2.403	-1.716
NIFMUN	1.418	1.395	1.387	1.406	1.38	1.416	1.391	1.404	1.411	1.373	4.431	7.81
NUQPOF	1.412	1.413	1.365	1.383	1.379	1.419	1.401	1.364	1.377	1.389	2.26	11.282
OGEROI	1.407	1.392	1.396	1.395	1.387	1.407	1.401	1.395	1.394	1.382	-0.621	-1.66
OGEROI	1.402	1.4	1.39	1.398	1.38	1.413	1.401	1.394	1.391	1.383	-1.054	2.687

PIRFIH	1.418	1.412	1.372	1.38	1.397	1.418	1.402	1.374	1.384	1.386	-9.13	10.835
PIRFON	1.422	1.417	1.372	1.385	1.388	1.429	1.41	1.381	1.382	1.389	-1.413	6.409
QEGXUV	1.405	1.386	1.371	1.4	1.376	1.408	1.366	1.377	1.403	1.369	10.494	-9.819
QEGXUV	1.415	1.369	1.369	1.395	1.37	1.418	1.399	1.36	1.394	1.373	-2.433	8.305
RAYXEX	1.427	1.37	1.357	1.407	1.378	1.396	1.384	1.38	1.398	1.377	2.352	2.702
RAYXIB	1.404	1.397	1.373	1.4	1.386	1.415	1.384	1.388	1.409	1.372	0.26	0.459
RAYXOH	1.42	1.387	1.38	1.405	1.378	1.411	1.394	1.38	1.405	1.382	0.238	2.349
RAYXUN	1.402	1.393	1.367	1.446	1.346	1.431	1.37	1.405	1.365	1.416	8.723	10.931
RAYYAU	1.404	1.396	1.391	1.409	1.376	1.413	1.395	1.383	1.396	1.38	4.563	0.577
RAYYEY	1.402	1.395	1.381	1.4	1.386	1.409	1.39	1.378	1.405	1.375	-0.637	-3.952
RHODON	1.406	1.415	1.382	1.413	1.395	1.4	1.419	1.376	1.388	1.381	7.837	8.023
RHODON	1.388	1.418	1.386	1.43	1.395	1.41	1.391	1.375	1.396	1.374	0.962	-3.881
ROFJUT	1.404	1.395	1.373	1.395	1.379	1.425	1.386	1.38	1.419	1.387	5.578	5.996
SAPQAB	1.391	1.408	1.357	1.423	1.401	1.406	1.406	1.399	1.394	1.394	0.745	0.707
SOHXAO	1.411	1.406	1.377	1.411	1.384	1.433	1.379	1.364	1.388	1.38	-2.745	5.99
SOHXAO01	1.417	1.393	1.393	1.397	1.384	1.412	1.397	1.384	1.404	1.385	0.67	2.273
SUQBOX	1.41	1.39	1.382	1.388	1.387	1.412	1.387	1.382	1.393	1.383	4.689	1.525
SUQCAK	1.414	1.387	1.38	1.389	1.387	1.413	1.384	1.381	1.392	1.382	-2.197	-2.714
TEVVOG	1.399	1.397	1.383	1.402	1.385	1.416	1.387	1.375	1.405	1.377	-0.555	-0.383
TIDCIS	1.408	1.392	1.363	1.383	1.38	1.42	1.386	1.355	1.378	1.383	-0.97	-5.338
TIDCIS	1.403	1.394	1.365	1.378	1.383	1.417	1.389	1.36	1.385	1.378	-1.9	3.559
TUPKUK	1.408	1.393	1.366	1.392	1.38	1.405	1.393	1.385	1.402	1.377	-3.511	-5.67
VANCET	1.414	1.394	1.376	1.39	1.391	1.418	1.39	1.378	1.392	1.388	0.124	-1.955
VERSIC	1.417	1.381	1.385	1.401	1.373	1.401	1.393	1.382	1.375	1.388	-1.753	8.178
VURHEV	1.441	1.32	1.376	1.394	1.392	1.373	1.429	1.443	1.414	1.372	18.655	-152.085
WOKYIH	1.411	1.394	1.378	1.386	1.383	1.415	1.393	1.373	1.398	1.382	4.215	1.117
WOKYIH	1.412	1.4	1.369	1.398	1.381	1.411	1.413	1.382	1.395	1.38	1.036	-2.994
XABSAX	1.412	1.393	1.376	1.393	1.387	1.415	1.392	1.375	1.392	1.368	-3.51	-0.245
XABSAX	1.414	1.395	1.369	1.398	1.382	1.416	1.391	1.367	1.393	1.373	-2.254	2.843
XABSAX	1.409	1.397	1.373	1.399	1.385	1.41	1.389	1.371	1.386	1.382	-1.704	1.432
XABSAX	1.404	1.407	1.375	1.394	1.382	1.402	1.393	1.377	1.391	1.376	-3.014	-5.578
XABSAX	1.397	1.41	1.367	1.391	1.381	1.415	1.387	1.375	1.397	1.38	-0.105	-3.036
XABSAX	1.42	1.411	1.374	1.394	1.389	1.405	1.385	1.374	1.393	1.367	-1.972	-3.125
YOKHIP	1.396	1.411	1.363	1.39	1.379	1.414	1.383	1.387	1.414	1.399	-0.291	1.092
ZISQUQ	1.39	1.407	1.346	1.419	1.41	1.417	1.388	1.373	1.387	1.384	-2.107	-2.4
VUMDIT	1.418	1.39	1.365	1.431	1.388	1.405	1.417	1.376	1.402	1.377	-8.364	-4.568
VUMDIT	1.426	1.398	1.371	1.427	1.389	1.403	1.422	1.381	1.384	1.381	-10.287	-8.622
YUKPUS	1.412	1.396	1.38	1.396	1.373	1.402	1.398	1.382	1.386	1.386	2.219	-2.458
